# Modulating vicarious tactile perception with transcranial electrical current stimulation

**DOI:** 10.1111/ejn.13699

**Published:** 2017-10-13

**Authors:** Natalie C. Bowling, Michael J. Banissy

**Affiliations:** ^1^ Department of Psychology Goldsmiths University of London London SE14 6NW UK

**Keywords:** mirror‐touch synaesthesia, primary somatosensory cortex, temporo‐parietal junction, transcranial random noise stimulation, transcranial direct current stimulation

## Abstract

Our capacity to share the experiences of others is a critical part of social behaviour. One process thought to be important for this is vicarious perception. Passively viewing touch activates some of the same network of brain regions as the direct experience of touch. This vicarious experience is usually implicit, but for some people, viewing touch evokes conscious tactile sensations (mirror‐touch synaesthesia). Recent work has attempted to induce conscious vicarious touch in those that do not normally experience these sensations, using transcranial direct current stimulation (tDCS). Anodal tDCS applied to primary somatosensory cortex (SI) was found to induce behavioural performance akin to mirror‐touch synaesthesia on a visuotactile interference task. Here, we conducted two experiments that sought to replicate and extend these findings by examining: (i) the effects of tDCS and high‐frequency transcranial random noise stimulation (tRNS) targeted at SI and temporo‐parietal junction (TPJ) on vicarious tactile perception, (ii) the extent to which any stimulation effects were specific to viewing touch to humans vs. inanimate agents and (iii) the influence of visual perspective (viewing touch from one's own vs. another's perspective) on vicarious perception. In Experiment 1, tRNS targeted at SI did not modulate vicarious perception. In Experiment 2, tDCS targeted at SI, but not TPJ, resulted in some modulation of vicarious perception, but there were important caveats to this effect. Implications regarding mechanisms of vicarious perception are discussed. Collectively, the findings do not provide convincing evidence for the potential to modulate vicarious tactile perception with transcranial electrical current stimulation.

## Introduction

Representing and understanding others’ experiences is crucial to facilitate social interactions and build interpersonal relationships. One process thought to be involved in this is vicarious perception – the ability to co‐represent the experiences of other people by matching the observed state onto representations of our own first‐hand experience. The degree of vicarious perception can be modulated by a range of top‐down factors (i.e. it can be socially and contextually embedded) such as our higher‐order beliefs about the person and the race or social status of the observed other. In this regard, vicarious perception has become a useful model for studying complex social abilities such as empathy (Bird & Viding, 2014).

One example of vicarious perception is vicarious touch. For instance, passively observing another person being touched appears to recruit overlapping brain regions as are involved in first‐hand experiences of touch, including activity in primary and secondary somatosensory cortex (Keysers *et al*., 2010). While vicarious tactile perception is common, there are important individual variability factors associated with this (Gillmeister *et al*., 2017). One source of variation is a distinction between those who experience conscious as opposed to unconscious vicarious tactile responses. In particular, for a small minority of individuals (<2%; Banissy *et al*., 2009) with mirror‐touch synaesthesia (MTS), seeing another person being touched elicits a conscious sensation of touch on their own body, as if they were being touched themselves (see Ward & Banissy, 2015 for review).

The reasons why some people experience conscious vicarious touch but others do not remain a topic of debate. One account (known as Threshold Theory; see Ward & Banissy, 2015 for review) assumes that the conscious sensation of touch arises from hyperactivity in somatosensory cortex when viewing touch to others. This activity is thought to boost vicarious responses above a threshold for conscious perception. In line with this, individuals with MTS demonstrate greater activation compared to controls in primary and secondary somatosensory cortices during the observation of touch (Blakemore *et al*., 2005; Holle *et al*., 2013). Further, there is some evidence suggesting that increasing cortical excitability in the somatosensory cortex of individuals that do not experience MTS can induce behavioural correlates of the experience, when viewing touch to others (Bolognini *et al*., 2013). In that study, participants were tested on an adapted version of a visuotactile interference task that had previously been shown to distinguish individuals with MTS from control participants (Banissy & Ward, 2007). This task requires participants to state the location of a tactile stimulus on their own body while simultaneously observing another person being touched. The felt touch can either be congruent (in the same spatial location) or incongruent (on the opposite side of the body) to the observed touch. For individuals with MTS, there are increased congruency effects, with incongruent trials producing longer reaction times and a greater number of errors consistent with their conscious vicarious touch (Banissy & Ward, 2007). Bolognini *et al*. report that greater congruency effects can be induced in non‐synaesthetes on this task by increasing somatosensory cortex excitability using transcranial direct current stimulation (tDCS): a tool that enables cortical excitability to be manipulated by passing a low amount of electric current between two electrodes placed on the scalp (Nitsche & Paulus, 2000). More specifically, participants showed increased congruency effects in their reaction times after tDCS targeted at the somatosensory cortex on the ipsilateral side to the tactile stimulus (and thus the contralateral side to the observed touch), when another hand was seen being touched, compared with an inanimate object (a lightbulb). Further, participants with higher self‐reported perspective taking (a subscale of the Interpersonal Reactivity Index measure of empathy; Davis, 1980) showed a greater effect of stimulation, indicating that individual difference factors might mediate the effects of tDCS on task performance. The results suggest that increased somatosensory cortex excitability underlies vicarious tactile experience, supporting a Threshold Theory account.

While the study from Bolognini *et al*. (2013) points to cortical excitability in the somatosensory system playing a pivotal role in vicarious tactile perception, there are a number of important questions that need to be clarified. For example, as the only control task used in the experiment involved touch to a lightbulb, it remains unclear whether the effects are specific to human touch or whether a human form physically and spatially congruent with the participant's own body is sufficient (e.g. dummy body parts). Previous behavioural research has also found that viewing touch to hands in a first‐ vs. third‐person perspective can influence task performance (Vandenbroucke *et al*., 2015), but whether visual perspective influences performance change following brain stimulation has not been studied. Additional work is therefore needed to (i) examine the replicability of findings indicating that increasing excitability within the somatosensory system can induce MTS in non‐synaesthete controls and (ii) consider how variations in stimulus presentation (e.g. animacy, perspective of viewed stimuli) contribute to previously reported effects.

Further, in addition to tDCS, other forms of electrical current stimulation have recently been used to modulate perceptual and cognitive task performance, for example with transcranial alternating current stimulation (Kanai *et al*., 2008; Marshall *et al*., 2006; Benwell *et al*., 2015; Janik *et al*., 2015) and with high‐frequency transcranial random noise stimulation (tRNS; Cappelletti *et al*., 2013; Fertonani *et al*., 2011; Snowball *et al*., 2013; Romanska *et al*., 2015). Of relevance to the current study is high‐frequency tRNS. As with tDCS, this method involves passing a weak electrical current to the brain via electrodes placed on the scalp, but tRNS differs in delivery and inducing neural change. While tDCS involves passing a homogenous current leading to a unilateral increase or decrease in brain excitability, tRNS involves passing an alternating current at a range of frequencies (from 100 to 640 Hz in high‐frequency tRNS) that results in a bilateral increase in cortical excitability (e.g. Terney *et al*., 2008). Comparisons of the two techniques suggest that high‐frequency tRNS may exert greater effects on changing cortical excitability (Vanneste *et al*., 2013), although different mechanisms of action may contribute to cortical excitability effects of tRNS and tDCS (Miniussi *et al*., 2013; Paulus *et al*., 2016). As yet, no study has examined if high‐frequency tRNS might be useful to modulate vicarious perception, but given that in some circumstances bilateral somatosensory cortex activity is likely to contribute to perceiving touch to other people, then this technique may offer a useful approach to examine the effect of increasing bilateral cortical excitability in the somatosensory cortices on tactile perception. With this in mind, in addition to re‐examining prior effects suggesting that tDCS targeted at the somatosensory cortex on the contralateral side to observed touch can induce MTS in non‐synaesthetes, we also sought to examine whether high‐frequency tRNS targeted at bilateral somatosensory cortices would have similar effects.

Although excitability within the somatosensory system is likely to be one mechanism contributing to how we perceive tactile events to others, there are other brain regions that are likely to play a key role. One set of mechanisms thought to be involved in vicarious perception relates to the ability to correctly distinguish and manipulate self‐relevant or other‐relevant representations. Appropriate levels of vicarious perception require enhancing representations of other people and inhibiting the representation of one's own affective state; however, to prevent excessive personal distress from another's negative affective state, it can also be adaptive to inhibit the representations of the other and enhance representations of the self (Cheng *et al*., 2007; Decety *et al*., 2010). In this regard, the interplay between mechanisms of vicarious perception and mechanisms of self‐other representation has been highlighted as a crucial interaction in understanding other people's experiences (Bird & Viding, 2014; Lamm *et al*., 2016; Ward & Banissy, 2015). Indeed, recent work suggests that training the ability to control self‐other representations can result in modulation of vicarious pain perception (de Guzman *et al*., 2016). TDCS targeted at the right temporo‐parietal junction (rTPJ) has been shown to increase the ability to control self‐other representations (Santiesteban *et al*., 2012) and modulate cognitive components of empathy for pain (Coll *et al*., 2017). To date, a single study has attempted to examine whether stimulating rTPJ can influence vicarious tactile perception. Vandenbroucke *et al*. (2016) repeated the visuotactile interference task used by Banissy & Ward (2007) and Bolognini *et al*. (2013), this time aiming to modulate performance following tDCS targeted at rTPJ. As increasing cortical excitability in rTPJ has been shown to improve self‐other control, the authors predicted that accuracy and reaction times would improve on the task following stimulation. However, this modulation was not found, in response to either viewed touch or pain (conflicting with Coll *et al*., 2017). One possible reason for this was that touch was always viewed to another human hand in a first‐person perspective, which could conceivably be viewed as belonging to the self. In Experiment 2, we sought to assess this possibility by assessing whether any effect of stimulation was specific to viewing touch to human vs. inanimate agents by including our dummy hand control task – a question that was not addressed in prior work.

The present experiments aimed to identify the contribution of body congruency and perception of animacy in modulating vicarious tactile perception following transcranial electrical stimulation. To study this, visuotactile interference tasks were administered in which touch is viewed to an object, to inanimate dummy hands and to human hands in either a first‐person or third‐person perspective, relative to the observer. In one study, we examined the impact of tRNS targeted at bilateral primary somatosensory cortices (SI), and in another, we examine the effects of tDCS (Experiment 2) targeted at right somatosensory cortex (rSI) and the right temporo‐parietal junction (rTPJ). Based on prior research, increasing cortical excitability in somatosensory brain regions was expected to increase vicarious tactile perception when viewing another person being touched.

## Materials and methods

### Participants

Twenty‐four healthy participants took part in both sessions of Experiment 1 (22F, 2M; 24 right‐handed; age 18–58 years, *M *=* *21.7, SD = 8.2). An additional 24 participants (16F, 8M; 23 right‐handed; age 20–29 years, *M *=* *23.2, SD = 2.6), who did not take part in Experiment 1, were recruited for Experiment 2. All participants gave fully informed consent and were paid £20 on completion of the study. All had normal or corrected‐to‐normal vision and met the required safety precautions to take part in electrical brain stimulation outlined by Bikson *et al*. (2009). Ethical approval was granted by the Department of Psychology at Goldsmiths, University of London.

### Transcranial current stimulation protocol

Experiment 1 comprised two stimulation conditions: active or sham high‐frequency tRNS delivered bilaterally to SI. Experiment 2 consisted of three conditions: active tDCS targeted unilaterally at right SI (rSI) or right TPJ (rTPJ), and sham stimulation. Both experiments had a within‐subjects design, with all participants completing the tasks under each stimulation condition, in separate sessions. Experimental sessions were scheduled 3–7 days apart to avoid practice effects, with the order of sessions counterbalanced between participants. Stimulation was delivered with two 5 × 5 cm saline‐soaked sponge electrodes and a constant‐current stimulator (NeuroComm, DC‐Stimulator Plus).

To target bilateral SI in Experiment 1, electrodes were placed 2 cm posterior to C3/C4, according to the 10–20 electroencephalography system (Herwig *et al*., 2003). High‐frequency tRNS was delivered offline, immediately prior to the tasks. As effects of offline tRNS have been shown to last up to one hour following 10 min of stimulation (Terney *et al*., 2008), this allowed a longer time window to complete the additional tasks administered in this experiment. The current was ramped up for 15 s to 1.5 mA based on the intensity used in prior work (Bolognini *et al*., 2013) and was followed by 10 min of stimulation, before ramping down again for 15 s. The sham protocol was identical to active stimulation, with the exception that the current was held constant for only 15 s before ramping down (although the electrodes were left in place for 10 min). This allowed the same initial mild scratching sensation to be experienced in the same location as during active stimulation (Ambrus *et al*., 2010; Fertonani *et al*., 2015).

To target rSI in Experiment 2, the anodal electrode was placed 2 cm posterior to C4, and for rTPJ, the anode was placed over CP6 (Herwig *et al*., 2003). A supraorbital reference on the contralateral hemisphere was used for both sites. For 50% of participants, the rSI site was used during sham, and for 50%, the rTPJ site was used. Active stimulation was delivered online for 20 min during completion of the tasks. As before, the current was ramped up for 15 s to 1.5 mA and then held constant for 20 min. Stimulation was terminated if participants completed both tasks in less than 20 min. In the sham session, stimulation was delivered for only 15 s (Gandiga *et al*., 2006; Nitsche *et al*., 2008; Poreisz *et al*., 2007). All aspects of the stimulation protocol were selected to match that used by Bolognini *et al*. (2013), aside from the placement of electrodes for rTPJ stimulation, which was guided by consensus in previous tDCS research (e.g. Santiesteban *et al*., 2012, 2015a; Vandenbroucke *et al*., 2016).

### Visuotactile interference tasks

**Figure 1:**
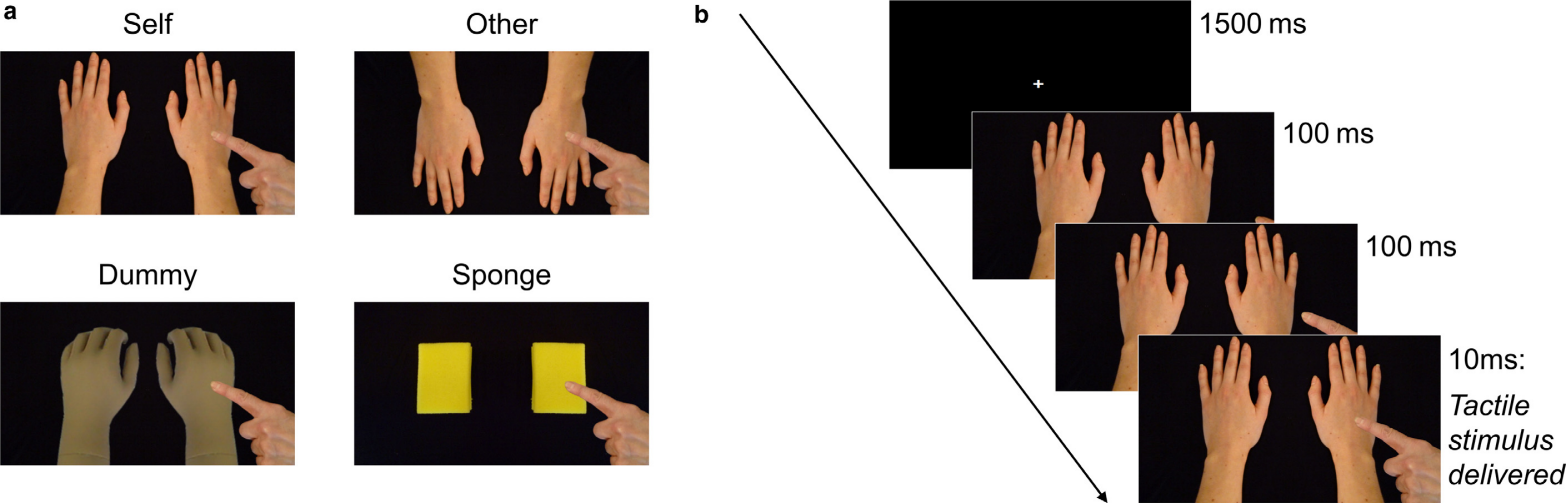
(a) Visual stimuli depicting the agent in the ‘self’, ‘other’, ‘dummy’ and ‘sponge’ tasks, and (b) Example trial structure from the ‘self’ task. [Colour figure can be viewed at http://www.wileyonlinelibrary.com/].

Participants completed visuotactile interference tasks in each experimental session of Experiments 1 and 2. For these, participants were required to state the location of a tactile sensation on their own hand, while simultaneously observing another agent (hand or object depending on task) being touched. Observed touch occurred either to another human hand in an egocentric body location (‘self’ task), an allocentric location (‘other’ task), to a dummy hand (‘dummy’ task) or to a sponge (‘sponge’ task). Visual stimuli are shown in Fig. 1a. In Experiment 1, all four tasks were administered in each session, and in Experiment 2, only the ‘self’ and ‘dummy’ tasks were completed. In each experiment, the order of tasks was counterbalanced between participants. The tactile stimulus was delivered using two miniature solenoid tappers attached to the dorsum of the participant's left and right hands with medical tape. A Dual Channel Solenoid Controller (MSTC3‐2; M & E Solve) was used to control the tappers.

The visuotactile interference task was adapted from Banissy & Ward (2007) and presented in E‐Prime 1.0 (Psychology Software Tools Inc., Pittsburgh, PA) using a 19” Hannspree monitor placed approximately 50 cm in front of the participant. For each trial, participants viewed three consecutive images displaying hands being approached and touched by an index finger (Fig. 1b). Observed touch was shown on the left, right or both sides. Each trial was preceded by a 1500 ms fixation cross. A tactile stimulus was delivered via the solenoid tappers attached to the hands 10 ms after the onset of observed touch. The observed touch then remained on the screen until the participant gave a response. The tactile stimulus was either delivered on the left, right or both sides, or there was no touch at all. On 60 trials, the felt touch was spatially congruent with the observed touch or flash, and on 60 trials, it was incongruent, and on 60 trials, there was no touch. Participants gave verbal responses using a voice key by stating ‘left’, ‘right’, ‘both’ or ‘none’, according to which location they felt the tactile stimulus. All participants were asked to respond as quickly and accurately as possible. The order of trials was pseudorandomized over three blocks. White noise was played through headphones during each trial to mask the noise of the solenoid tappers.

### Procedure

There were two sessions to Experiment 1, one for sham and one for SI stimulation. Stimulation was delivered offline, immediately prior to the visuotactile tasks. The order of the four tasks (‘self’, ‘other’, ‘dummy’ and ‘sponge’) was counterbalanced between participants, and it took no more than 40 min to complete all four tasks. During the tasks, participants were instructed to place their hands flat on the desk in front of them, in the same manner as the visual stimuli shown in the ‘self’ task (Fig. 1a), and to keep their eyes focused on the screen. Participants completed items from the QMTS (Bolognini *et al*., 2013) at the end of both sham and active sessions, and the IRI (Davis, 1980) at the end of the sham session.

Experiment 2 comprised three sessions: rSI, rTPJ and sham stimulation. In this procedure, tDCS was delivered online, while the two visuotactile interference tasks (‘self’ and ‘dummy’) were completed. Online tDCS was used to replicate prior work (Bolognini *et al*., 2013). It took no more than 20 min to complete both tasks. As in Experiment 1, participants completed the QMTS (Bolognini *et al*., 2013) at the end of every session, and the IRI (Davis, 1980) at the end of the sham session.

### Self‐report measures

A series of self‐report measures were also completed in each experiment:

1. Self‐reported mirror‐touch synaesthesia (adapted from Banissy *et al*., 2009): at the beginning of the first session of the experiment, participants were asked ‘Do you experience touch sensations on your own body when you see them on another person's body?’, and could respond on a 5‐point Likert scale from ‘Strongly Disagree’ to ‘Strongly Agree’. Responses were coded from −2 to +2, where a positive score indicates self‐reported experience of synaesthesia. This was completed to screen participants for potential mirror‐touch synaesthesia.
2. Questionnaire of Mirror‐Touch Synaesthesia (QMTS; from Bolognini *et al*., 2013): each participants’ experience during the interference tasks was assessed using items 1, 4 and 5 from the Questionnaire of Mirror‐Touch Synaesthesia (QMTS) used by Bolognini *et al*. (2013). This was administered at the end of each session. Participants were required to state the extent to which they agreed with six statements using a 5‐point scale ranging from ‘Strongly Disagree’ to ‘Strongly Agree’. Items comprised: (i) ‘I felt that I was touched when I saw the human hand/dummy hand being touched’, (ii) ‘Seeing the human hand/dummy hand being touched made it difficult to localize the actual touch’ and (iii) ‘The observed touch to the human hand/dummy hand appeared to be very intense’. Again, scores for each item were coded from −2 to +2 during data analysis.
3. Self‐reported Empathy (Davis, 1980): the 28‐item Interpersonal Reactivity Index (IRI) was used to assess self‐report trait empathy. This questionnaire asks participants to indicate the extent to which they agree with each of 28 statements, such as ‘I often have tender, concerned feelings for people less fortunate than me’, using a 5‐point scale ranging from ‘Does not describe me well’ to ‘Describes me very well’. Total scores range from 0–112, with a higher score indicating higher trait empathy. Scores can also be clustered into four subscales, reflecting ‘Fantasy’, ‘Perspective Taking’, ‘Empathic Concern’ and ‘Personal Distress’ Davis (1980) reports an acceptable internal consistency for each of the subscales (α = 0.70–0.78).

## Results

### Experiment 1: Effects of high‐frequency tRNS targeted at bilateral somatosensory cortex on vicarious tactile perception

Prior to analyses, data were trimmed for each participant to exclude any reaction time (RT) that fell two standard deviations above or below the mean for each task and stimulation condition. This resulted in 5.0% of data removal. Two participants were identified as significant outliers based on Grubb's test calculations on RTs and were excluded prior to analysis. This resulted in the following demographic characteristics of the sample: 20 female, two male; age 18–58 years, *M *=* *22.0, SD = 8.5. This did not differ significantly from the sample recruited by Bolognini *et al*. (2013) in terms of age (*t*_52_ = 0.84, *P *=* *0.41), but did differ in the proportion of males and females (χ^2^_1_ = 6.97 (*n *=* *54), *P *=* *0.01), with fewer males participating in our experiment compared with the previous sample.

It was also necessary to calculate individual spatial reference frames for all participants, in order to categorize trials as either congruent or incongruent. There are two potential reference frames that can be adopted during the allocentric task: (i) anatomically congruent (where viewing touch to a left hand is matched to participants’ left hand) or (ii) specular congruence (where viewing touch to a left hand is matched to a participants’ right hand). Congruency was defined for each participant depending on whichever mapping gave the largest congruency score in the sham condition of the ‘other’ task, and this was used in analyses throughout a given participant (i.e. if the RT was longer for a specular mapping in the sham task, then the participant was classified as a specular mapper, and vice versa). This analysis revealed 20 specular and 2 anatomical mappers.

#### Individual differences in trait empathy and sham task performance

To identify whether reaction times on the vicarious tactile perception tasks were related to individual differences in empathy, Pearson's correlation analyses were carried out between scores on the IRI subscales and RTs on each of the four tasks in the sham condition. A statistically significant correlation was found between scores on the ‘perspective taking’ subscale and RTs on congruent trials of the ‘self’ task, in sham stimulation conditions (*r*_20_ = −0.43, *P *=* *0.05). The negative correlation indicates that higher perspective taking ability facilitated tactile detection on the ‘self’ task when observed touched was spatially congruent with touch felt on the hand.

#### Effects of high‐frequency tRNS on task performance

**Figure 2:**
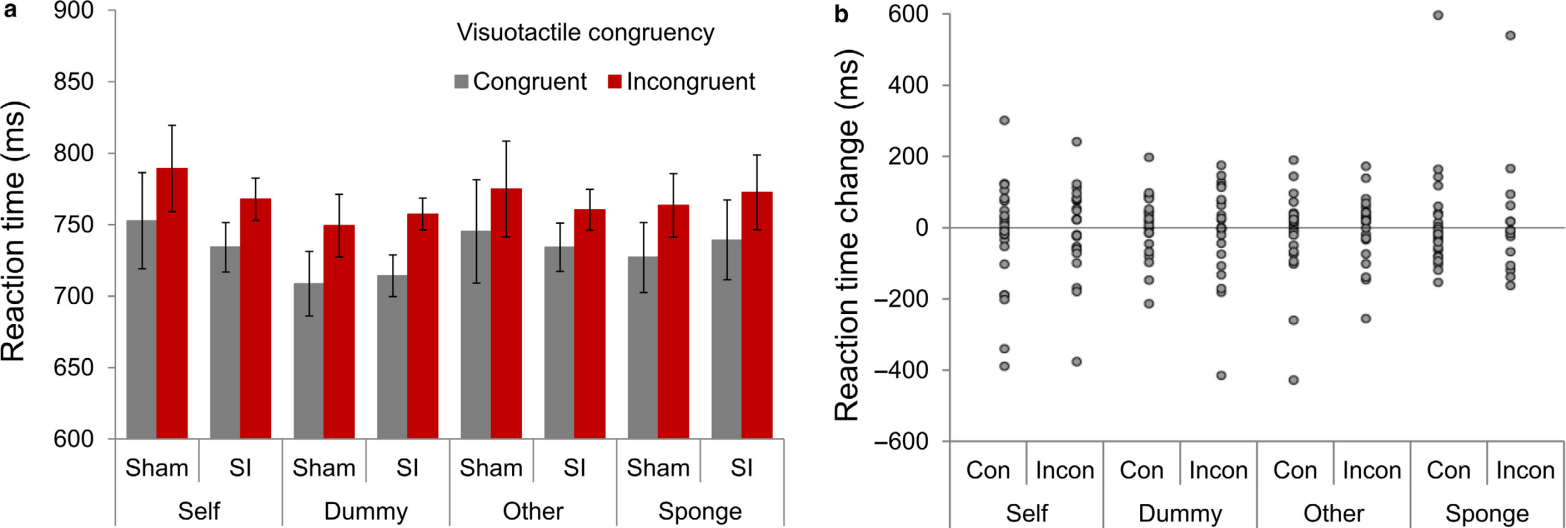
(a) Mean reaction times for congruent and incongruent trials on each of the four visuotactile interference tasks following sham or tRNS targeted at SI. Significant congruency effects were found on all tasks. (b) Individual stimulation effects (reaction time in tRNS condition – reaction time in sham condition) for congruent and incongruent trials on each task. Con, Congruent; Incon, Incongruent. Error bars represent ±1 SEM. [Colour figure can be viewed at http://www.wileyonlinelibrary.com/].

To examine the effects of high‐frequency tRNS on task performance, a 4 (Task) × 2 (Stimulation) × 2 (Congruency) repeated‐measures anova was carried out to identify the effects of task (‘self’/’dummy’/’other’/'sponge’), tRNS condition (sham/SI) and congruency (congruent/incongruent) on reaction times. There was a significant main effect of Congruency (*F*_1,21_ = 56.24, *P *<* *0.01, ɳ_p_^2^ = 0.73), with longer reaction times on incongruent trials than congruent trials. However, there was no significant main effect of Task (*F*_3,63_ = 1.48, *P *=* *0.23, ɳ_p_^2^ = 0.07), or Stimulation condition (*F*_1,21_ = 0.03, *P = *0.86, ɳ_p_^2^ < 0.01) or interactions between any of the three factors (*P*s > 0.12). As this analysis was of particular interest for assessing the replicability of previously reported effects (Bolognini *et al*., 2013), we supplemented the results with a Bayesian approach. A Bayes factor anova using default priors in JASP (Wagenmakers *et al*., 2017) found strongest evidence for a model containing a main effect of Congruency only (BF_M_ = 48.94). The Bayes factor for inclusion of the crucial interaction between Stimulation and Congruency (based on Bayesian model averaging, see Etz & Wagenmakers, 2017) provided evidence against inclusion in the model (BF_Inclusion_ = 0.04). In this regard, high‐frequency tRNS targeted over SI did not differentially modulate vicarious tactile perception relative to sham stimulation (Fig. 2).

To further assess any effects of stimulation, we also analysed scores on QMTS self‐report measure that asked about potential MTS experiences at the end of each task type. To do this, we conducted a 4 (Task) × 2 (Stimulation) anova on responses to overall scores. Again this identified a significant main effect of Task (*F*_3,60_ = 9.86, *P *<* *0.01, ɳ_p_^2^ = 0.33), but no significant main effect of Stimulation condition (*F*_1,20_ = 0.34, *P *=* *0.57, ɳ_p_² = 0.02), or interaction between Stimulation and Task (*F*_3,60_ = 0.53, *P *=* *0.66, ɳ_p_^2^ = 0.03). Bonferroni‐corrected post hoc *t*‐tests demonstrated that the main effect of task was due to significantly lower scores (indicating reduced vicarious sensation) for the ‘dummy’ task compared with the ‘self’ (*t*_20_ = 3.46, *P *<* *0.01, Cohen's *d *=* *0.90) or ‘other’ task (*t*_20_ = 3.19, *P *<* *0.01, Cohen's *d *=* *0.80), and for the ‘sponge’ task compared with the ‘self’ (*t*_20_ = 3.43, *P *<* *0.01, Cohen's *d *=* *1.01) and ‘other’ tasks (*t*_20_ = 3.05, *P *<* *0.01, Cohen's *d *=* *0.88). No significant difference in scores was found between the ‘dummy’ and ‘sponge’ tasks (*t*_20_ = 1.09, *P *=* *0.29, Cohen's *d *=* *0.27), or the ‘self’ and ‘other’ tasks (*t*_20_ = 1.96, *P *=* *0.06, Cohen's *d *=* *0.48). Means are displayed in Table S1. The pattern of results indicates a greater tendency towards conscious vicarious tactile perception on the tasks in which touch was viewed to another human hand, compared with an inanimate object, but that this was not modulated by high‐frequency tRNS targeted at SI.

#### Individual differences in trait empathy and effects of tRNS on task performance

While tRNS stimulation targeted at SI did not significantly alter task performance at the group level, based on our results above (Section 2.2.1) and prior research (Bolognini *et al*., 2013) there was reason to predict that the effects of tRNS on task performance may interact with individual differences in trait empathy (specifically the perspective taking subscale of the IRI). To examine this prediction, we conducted a series of correlations between scores on perspective taking subscale of the IRI and stimulation effect scores (the difference between congruency effects in the active tRNS and sham condition) on the ‘self’, ‘other’, ‘dummy’ and ‘sponge’ tasks. This revealed no significant relationships (Table S2).

### Experiment 2: Effects of tDCS targeted at right hemisphere SI and TPJ on vicarious tactile perception

Prior to analyses, data were trimmed for each participant to exclude any reaction time (RT) that fell two standard deviations above or below the mean for each task and stimulation condition. This resulted in 4.6% of data removal. One participant was also excluded prior to the analysis, as they were identified as a significant outlier based on Grubb's test calculations on RTs. This resulted in the following demographic characteristics of the sample: 15 female, 8 male; age 20–29 years, *M *=* *23.3, SD = 2.7. This did not differ significantly from the sample recruited by Bolognini *et al*. (2013) in terms of age (*t*_53_ = 0.63, *P *=* *0.53) or gender (χ²_1_ = 0.38, (*n *=* *55), *P *=* *0.54).

#### Individual variability in trait empathy and task performance in sham condition

Scores on the IRI (Davis, 1980) were first correlated against RTs in each of the stimulation, task and congruency conditions. Unlike Experiment 1, no significant correlations were found.

#### Effects of tDCS on task performance

To examine whether active or sham tDCS to rSI or rTPJ resulted in differential effects on performance, a 3 (Stimulation Type) × 2 (Task) × 2 (Congruency) × 2 (Location) repeated‐measures anova was carried out to assess the effects of tDCS stimulation (rSI/rTPJ/sham), task (‘self’/’dummy’), congruency (congruent/incongruent) and location of the tactile stimulus (left/right) on RTs.

**Figure 3:**
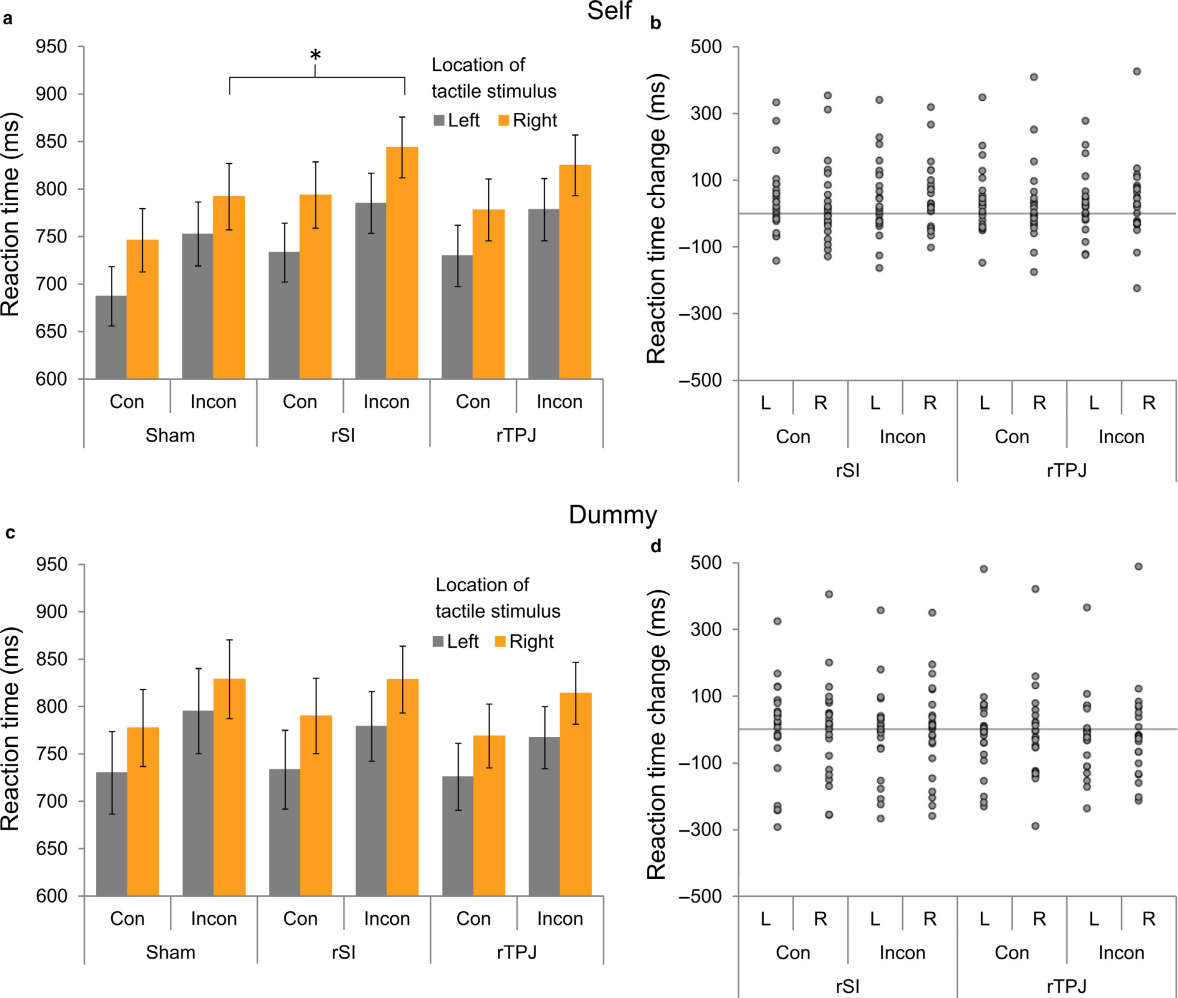
(a) Mean reaction times for each trial type on the ‘self’ task during sham conditions and tDCS targeted at rSI and rTPJ. A significant increase in RT was observed following SI stimulation, for incongruent trials where observed touch was contralateral to the stimulation site. (b) Individual stimulation effects (reaction time in tDCS condition – reaction time in sham condition) for each trial type on the ‘self’ task. (c) Mean reaction times for each trial and stimulation type on the ‘dummy’ task. No significant effects of stimulation were observed on this task. (d) Individual stimulation effects for each trial type on the ‘dummy’ task. Con, Congruent; Incon, Incongruent. L, Left; R, Right. Error bars represent ±1 SEM. *P < 0.05. [Colour figure can be viewed at http://www.wileyonlinelibrary.com/].

The analysis revealed a significant main effect of Congruency (*F*_1,22_ = 45.93, *P *<* *0.01, ɳ_p_² = 0.68) and Location (*F*_1,22_ = 41.29, *P *<* *0.01, ɳ_p_² = 0.65) on RTs, with participants taking longer to respond when the tactile stimulus was incongruent with the visual stimulus, and when the tactile stimulus was presented on the right hand rather than the left. Main effects of Stimulation (*F*_2,44_ = 0.47, *P *=* *0.63, ɳ_p_² = 0.02) and Task (*F*_1,22_ = 0.30, *P *=* *0.59, ɳ_p_² = 0.01) were not significant. The interaction between Stimulation and Task was significant (*F*_2,44_ = 3.37, *P *=* *0.04, ɳ_p_² = 0.13). Post hoc *t*‐tests demonstrate a trend towards significance following rSI stimulation on the ‘self’ task (*t*_22_ = 2.05, *P *=* *0.05, Cohen's *d *=* *0.43), but not the ‘dummy’ task (*t*_22_ = 0.01, *P *=* *0.99, Cohen's *d *<* *0.01), and no significant effects of rTPJ stimulation on either the ‘self’ (*t*_22_ = 1.54, *P *=* *0.14, Cohen's *d *=* *0.32) or ‘dummy’ task (*t*_22_ = 0.46, *P *= 0.65, Cohen's *d *=* *0.10). Together, this indicates that participants were slower to respond on the ‘self’ task following rSI stimulation. Crucially, the interaction between Stimulation and Congruency did not reach significance (*F*_2,44_ = 2.51, *P *=* *0.09, ɳ_p_² = 0.10). As for Experiment 1, this analysis was supplemented with a Bayesian anova, which found strongest evidence for a model containing main effects of Congruency and Location only (BF_M_ = 153.94). The Bayes factor for inclusion of the crucial interaction between Stimulation and Congruency provided evidence against inclusion in the model (BF_Inclusion_ = 0.01). In this regard, we did not find evidence to suggest a significantly different pattern of results between the size of congruency effects across the stimulation conditions (Fig. 3).

Despite the lack of interaction with regard to reaction time differences, there was rationale to consider a slightly less conservative approach to analysis given prior predictions regarding SI effects based on the previous study (e.g. Bolognini *et al*., 2013). With this in mind, in order to further identify whether the results presented by Bolognini *et al*. (2013) had been replicated, a series of independent *t*‐tests were carried out to assess whether RTs in each task, congruency, side and stimulation condition (rSI or rTPJ) significantly differed from sham. We also conducted Bayes factor *t*‐tests for these comparisons in JASP. This revealed a significant increase in RT on incongruent trials of the ‘self’ task during rSI stimulation relative to sham, when touch was felt on the right and observed on the left (contralateral) side to the stimulation (*t*_22_ = 2.31, *P *=* *0.02, Cohen's *d *=* *0.51), although we note that this would not survive correction for multiple comparison. While the Bayes factor supported the effect of tDCS in this condition (BF_10_ = 2.40), it was large enough to provide only anecdotal evidence (Wagenmakers *et al*., 2017). Stimulation effects were not significant when observed touch was ipsilateral to stimulation, although there was a trend in this direction (*t*_22_ = 1.97, *P *=* *0.06, Cohen's *d *=* *0.41, BF_10_ = 1.12). A similar trend was found on congruent trials of the ‘self’ task, when observed touch was on the left (contralateral) side to both rSI (*t*_22_ = 2.05, *P *=* *0.05, Cohen's *d *=* *0.43, BF_10_ = 0.45) and rTPJ (*t*_22_ = 2.02, *P *=* *0.06, Cohen's *d *=* *0.42, BF_10_ = 0.49) stimulation, relative to sham. No further comparisons reached significance (*P*s > 0.12, BF_10_ < 1.21). In this regard, although we observe some evidence pointing towards data that is consistent with prior work suggesting that increasing cortical excitability in SI can modulate the degree of vicarious tactile perception, the present data struggle to provide strong evidence (e.g. differential effects across stimulation sites as supported by a top level anova) to support this claim. We also do not find evidence that rTPJ stimulation influences vicarious tactile perception.

As with Experiment 1, we also assessed whether there was any effect of stimulation on the QMTS self‐report measure that asked about potential MTS experiences at the end of each task type. A further 2 (Task) × 3 (Stimulation) anova identified a main effect of task on the QMTS, with higher ratings of conscious vicarious experience on the ‘self’ task than the ‘dummy’ task (*F*_1,21_ = 13.57, *P *<* *0.01, ɳ_p_² = 0.39). However, there was no significant main effect (*F*_1,21_ = 0.63, *P *=* *0.54, ɳ_p_² = 0.03) or interaction (*F*_2,42_ = 0.65, *P *=* *0.53, ɳ_p_² = 0.03) with stimulation condition, indicating that conscious vicarious experience during the tasks did not change substantially between sessions (means are displayed in Table S3). This is consistent with the lack of effect of tDCS on congruency reaction times that we report above and the lack of effect of active high‐frequency tRNS in Experiment 1.

#### Individual differences in trait empathy and effects of tDCS on task performance

As with Experiment 1, we also assessed whether the effects of tDCS on task performance may interact with individual differences in trait empathy (specifically the perspective taking subscale of the IRI). To examine this prediction, we conducted a series of correlations between scores on perspective taking subscale of the IRI and stimulation effect scores (the difference between congruency effects in the active tDCS and sham conditions) on the ‘self’ and ‘dummy’ tasks. This revealed no significant association between perspective taking and stimulation effects (Table S4).

## General discussion

The present studies aimed to build on past evidence suggesting that vicarious responses to touch may be enhanced by increasing excitability of SI. Experiment 1 sought to extend prior findings regarding the effects of tDCS in vicarious tactile perception (Bolognini *et al*., 2013) by determining whether vicarious responses can also be enhanced following high‐frequency tRNS targeted at bilateral SI. The findings from Experiment 1 indicated that active compared to sham high‐frequency tRNS targeted at SI does not modulate vicarious tactile perception on tasks assessing visuotactile interference effects when observing touch to humans (from an allocentric and egocentric perspective) or objects. We also did not find any significant differences between active SI and sham tRNS conditions on self‐reported mirror‐touch synaesthesia experiences across the tasks or relationship between levels of perspective taking and performance change following stimulation (as was found in Bolognini *et al*., 2013).

One reason why we may have observed differences between our results in Experiment 1 and those using tDCS in prior work (e.g. Bolognini *et al*., 2013) could relate to the mechanism of action of the different types of stimulation. Prior work suggests that high‐frequency tRNS and tDCS may influence brain excitability via different mechanisms (Terney *et al*., 2008; Miniussi *et al*., 2013; Paulus *et al*., 2016). As a consequence, we conducted a second experiment, in a new group of participants, where we used the same brain stimulation procedure (i.e. using matched tDCS parameters) as that used in prior work in attempt to determine whether we were able to replicate the prior pattern of data. In addition, we also sought to extend prior work by considering extra questions related to vicarious tactile perception. In particular, we sought to examine whether tDCS targeted at the rTPJ, in addition to SI, may influence vicarious perception. Our rationale for examining the effect of stimulating the rTPJ was to assess whether modulating a brain region linked to self‐other control may also modulate vicarious tactile perception (based on the suggested role of self‐other control in vicarious perception, e.g. Ward & Banissy, 2015; de Guzman *et al*., 2016). While we were able to find some evidence to support the claim that increasing unilateral cortical excitability in rSI with anodal tDCS was able to increase vicarious tactile perception in typical adults, this was only apparent when using liberal statistical thresholds. The overall pattern of data struggles to provide convincing evidence for the potential to modulate vicarious response with unilateral tDCS targeted at right SI in a task and site specific manner. We also did not find evidence that tDCS targeted at rTPJ could modulate vicarious tactile perception.

The present findings conflict with a prior tDCS study, which suggests that tDCS targeted at SI can lead to greater vicarious tactile perception and induce behavioural performance consistent with that found in mirror‐touch synaesthetes (Bolognini *et al*., 2013). In that study, the authors claim to induce behavioural performance consistent with individuals that experience mirror‐touch synaesthesia following anodal tDCS to somatosensory regions. Although we used a similar task and identical stimulation parameters, we were not able to clearly replicate this pattern of data. We did find some evidence to support this account in our experiment using tDCS targeted at right SI, but this relied on uncorrected statistical analyses and was not significantly different to the pattern of data following sham or rTPJ stimulation.

Although stimulation parameters were the same between studies in Experiment 2, subtle differences in procedure and individual variability in responsiveness of tDCS may explain this discrepancy. With regard to procedural differences, it is of note that we included an additional brain stimulation condition and trials compared to Bolognini *et al*. We also used our own stimuli and task (Banissy & Ward, 2007) and a different control task involving dummy hand stimuli as opposed to light bulb stimuli that were used previously. While it seems unlikely that this should decrease the likelihood of finding an effect, it is possible that these subtle variations may have contributed to the different pattern of data between the studies. Another possible reason for the discrepancy between the studies may be individual variability in responsiveness of tDCS. Several studies now point to the importance of individual variation in tDCS responsiveness, with differential effects being reported in other domains (i.e. not vicarious perception studies) according to baseline ability (e.g. Tseng *et al*., 2012; Hsu *et al*., 2014), traits (e.g. Sarkar *et al*., 2014), baseline level of neurophysiological state (e.g. Fresnoza *et al*., 2014; Labruna *et al*., 2016), gender (e.g. Kuo *et al*., 2006; Chaieb *et al*., 2008), age (e.g. Ross *et al*., 2011; Moliadze *et al*., 2015) and anatomy (e.g. Datta *et al*., 2012; Opitz *et al*., 2015). While the age and gender of our participants are consistent with Bolognini *et al*. in Experiment 2, we cannot be sure on all other individual difference factors that may distinguish our sample from the participants used in the prior study. We did consider some potentially relevant individual differences in our current study (e.g. trait empathy), but did not find that these modulated stimulation effects in a systematic fashion. Future work should more closely examine how individual variability may influence changes in vicarious tactile perception following tDCS targeted at somatosensory related areas.

The lack of significant increase in vicarious tactile perception following high‐frequency tRNS or tDCS targeted at somatosensory regions also contradicts predictions based on a Threshold Theory account of mirror‐touch synaesthesia, which suggests that increased baseline excitability in somatosensory regions may boost vicarious responses to observed touch over a threshold for conscious perception (see Ward & Banissy, 2015 for review). In both of our experiments, no strong evidence for modulation in conscious vicarious perception was found, either behaviourally or in self‐reported experience, when excitability was increased in SI. Past research has identified structural brain differences associated with MTS that extend outside of the somatosensory system (Holle *et al*., 2013), suggesting a potentially contrasting neural profile between individuals with and without MTS. For this reason, it may be wrong to assume that this unique perceptual experience can be induced in controls.

In line with the results of previous research (Vandenbroucke *et al*., 2016), enhancing excitability of rTPJ with tDCS also did not significantly modulate vicarious tactile perception. This region has previously been linked with self‐other control mechanisms, and stimulation of rTPJ with tDCS has been shown to improve the ability to accurately switch between representations of self and others, according to task demand (Santiesteban *et al*., 2012, 2015a). Individuals with MTS have been shown to have deficits in the ability to control self‐other representations when there is a need to inhibit others and enhance the self (Santiesteban *et al*., 2015b). Further, training typical adults to become better able to control self‐other representations can lead to modulation of vicarious pain perception, although the neural locus of how self‐other control training contributes to this effect has not been investigated (de Guzman *et al*., 2016). With this in mind, it is perhaps surprising that tDCS targeted at rTPJ did not improve the ability to inhibit vicarious responses to observed touch to another person, when responding to felt touch on the participant's own hands. It should be noted that the tDCS parameters in the present study differed from those used in previous experiments modulating activity of rTPJ with tDCS. For instance, prior work examining self‐other representation using tDCS targeted at rTPJ has stimulated offline at 1 mA using 5 × 7 cm electrodes (e.g. Santiesteban *et al*., 2012, 2015a; Sowden *et al*., 2015) or 5 × 7 cm and 10 × 10 cm electrodes (e.g. Liepelt *et al*., 2016). Similarly, Coll *et al*. (2017) used 2 mA tDCS with 5 × 7 cm electrodes to assess vicarious pain perception. Our decision to use a protocol involving online stimulation at 1.5 mA using 5 × 5 cm electrodes was selected to match that in the rSI stimulation condition (which was selected to replicate prior work – Bolognini *et al*., 2013). The differences between our rTPJ stimulation montage and those used in other studies may have affected the degree of modulation of rTPJ compared with past research. This could account for the lack of influence of rTPJ stimulation on vicarious perception in Experiment 2. Additionally, the degree to which self‐other control is pivotal to the particular tasks used is another an important consideration. Participants were instructed to respond with the location where they felt touch on their own hands, but were not explicitly told to inhibit the touch they saw on the screen (i.e. there were no explicit self‐other control demands). It is possible that when viewing hands from an egocentric perspective, the hands are represented as part of the self rather than other. In this case, there are fewer requirements to control self‐other representations. In future, it would be interesting to consider whether these mechanisms can be engaged to a greater extent by manipulating task instructions and design.

It is also important to consider how the present findings relate to the broader literature regarding the role of sensorimotor contributions to social perception. For instance, recent evidence has indicated that a range of social perception abilities are linked with sensorimotor cortex activity (e.g. Adolphs *et al*., 2000; Pourtois *et al*., 2004; Pitcher *et al*., 2008; Banissy *et al*., 2010; Keysers *et al*., 2010; Jacquet & Avenanti, 2015; Paracampo *et al*., 2016; Valchev *et al*., 2017). Several of these have used non‐invasive brain stimulation to show changes in social perception skills following sensorimotor cortex stimulation relative to appropriate control conditions (e.g. baseline, control brain stimulation conditions). There have, however, been few published replication attempts for these studies. The present study, together with the evidence of large inter‐individual differences in the effects of non‐invasive brain stimulation (e.g. Ridding & Ziemann, 2010; Hsu *et al*., 2016; Fertonani & Miniussi, 2017), calls for more systematic investigations and replications in this area.

While we did not observe stimulation effects, there were some behavioural effects of note. For example, the degree of vicarious touch perception was associated with self‐reported perspective taking when viewing spatially congruent touch to another human hand (vs. a dummy hand or object). Previous research has shown a positive correlation between perspective taking scores and activation in SI (Schaefer *et al*., 2012), as well as amplitudes of somatosensory‐evoked potentials (Martínez‐Jauand *et al*., 2012) when observing touch. Our results are in line with these findings. Despite this association between perspective taking and vicarious tactile perception, this factor was not found to interact with the effects of tDCS or high‐frequency tRNS on task performance in the present studies.

In summary, across two studies we do not find clear evidence that increasing cortical excitability in somatosensory regions of typical younger adult participants leads to differential changes in vicarious tactile perception from sham stimulation (Experiments 1 and 2) or stimulation to the rTPJ (Experiment 2). These findings conflict with prior results and threshold‐based accounts of individual differences in vicarious perception.

## Conflict of interest

The authors declare no conflict of interests.

## Author contributions

Both authors developed the experimental design and materials. NB carried out data collection and analysis. Both authors contributed to interpretation of the data and writing the manuscript.

## Data accessibility

Data sets are available on request from the corresponding author.

**Abbreviations**
IRI: interpersonal reactivity index
MTS: mirror‐touch synaesthesia
QMTS: questionnaire of mirror‐touch synaesthesia
rSI: right primary somatosensory cortex
rTPJ: right temporo‐parietal junction
RT: reaction time
SI: primary somatosensory cortex
tDCS: transcranial direct current stimulation
TPJ: temporo‐parietal junction
tRNS: transcranial random noise stimulation

## Supporting information

Table S1 Mean scores and standard deviations for QMTS items, following each tRNS stimulation session in Experiment 1.
Table S2 Correlations between Perspective Taking and effects of high frequency tRNS targeted at SI on the ‘self’, ‘other’, ‘dummy’, and ‘sponge’ tasks, in Experiment 1.
Table S3 Mean scores and standard deviations for QMTS items, following each tDCS stimulation session in Experiment 2.
Table S4 Correlations between Perspective Taking and effects of tDCS targeted at rSI or rTPJ on the ‘self’ and ‘dummy’ tasks, in Experiment 2.
