# Supplementary material for: Modulating vicarious tactile perception with transcranial electrical current stimulation
Source: Eur J Neurosci. 2017 Oct 13;46(8):2355–64. doi: 10.1111/ejn.13699 (PMC5900887; doi:10.1111/ejn.13699)
Supplement: Supplementary file 2 — Table S1 Mean scores and standard deviations for QMTS items, following each tRNS stimulation session in Experiment 1. Table S2 Correlations between Perspective Taking and effects of high frequency tRNS targeted at SI on the ‘self’, ‘other’, ‘dummy’, and ‘sponge’ tasks, in Experiment 1. Table S3 Mean scores and standard deviations for QMTS items, following each tDCS stimulation session in Experiment 2. Table S4 Correlations between Perspective Taking and effects of tDCS targeted at rSI or rTPJ on the ‘self’ and ‘dummy’ tasks, in Experiment 2. [file EJN-46-2355-s002.pdf]

Supplemental Tables

Supplemental Table 1: Mean scores and standard deviations for QMTS items, following each tRNS stimulation session in Experiment 1. Possible scores range from -6 to +6, with higher scores indicating greater self-reported vicarious tactile perception

	Active SI tRNS		Sham tRNS	
Visuotactile Interference Task	*M*	*SD*	*M*	*SD*
Self	-2.05	2.85	-1.59	3.32
Other	-2.23	2.71	-2.14	3.14
Dummy	-3.10	2.21	-3.05	2.28
Sponge	-3.27	1.88	-3.23	1.90

N = 22

Supplemental Table 2: Correlations between Perspective Taking and effects of high frequency tRNS targeted at SI on the 'self', 'other', 'dummy', and 'sponge' tasks, in Experiment 1. Perspective Taking was not associated with stimulation effects on any of the four tasks.

Visuotactile Interference Task	Perspective Taking
Self	-.18
Other	.14
Dummy	.04
Sponge	-.19

N = 22

Supplemental Table 3: Mean scores and standard deviations for QMTS items, following each tDCS stimulation session in Experiment 2. Possible scores range from -6 to +6, with higher scores indicating greater self-reported vicarious tactile perception.

	Active rSI tDCS		Active rTPJ tDCS		Sham tDCS	
Visuotactile Interference Task	*M*	*SD*	*M*	*SD*	*M*	*SD*
Self	-1.57	2.45	-1.23	3.01	-1.74	2.40
Dummy	-2.78	2.15	-3.00	2.25	-3.17	2.08

N = 23

Supplemental Table 4: Correlations between Perspective Taking and effects of tDCS targeted at rSI or rTPJ on the 'self' and 'dummy' tasks, in Experiment 2. Perspective Taking was not associated with stimulation effects in either task or stimulation condition.

Stimulation Site	Visuotactile Interference Task	Perspective Taking
rSI		
	Self	-.09
	Dummy	-.03
rTPJ		
	Self	-.15
	Dummy	.25

N = 22
