# Supplementary material for: Modulating vicarious tactile perception with transcranial electrical current stimulation
Source: Eur J Neurosci. 2017 Oct 13;46(8):2355–64. doi: 10.1111/ejn.13699 (PMC5900887; doi:10.1111/ejn.13699)
Supplement: Supplementary file 1 [file EJN-46-2355-s001.pdf]

EJN European Journal of Neuroscience FENS Federation of European Neuroscience Societies

Modulating vicarious tactile perception with transcranial electrical current stimulation

Natalie C. Bowling & Michael J. Banissy

Review timeline:

Submission date:	06 June 2017
Editorial Decision:	03 July 2017
Revision received:	27 July 2017
Accepted:	24 August 2017

Editor: Gregor Thut

1st Editorial Decision 03 July 2017

Dear Dr. Bowling,

Your manuscript was reviewed by two external reviewers and the process was handled by one of our Section Editors, Prof. Gregor Thut. Both reviewers commend your work for its rigor and high standard, and raise moderate to minor points for consideration, including additional statistics, analysis, Figures and discussion points. We believe you will be able to address these issues, and invite you to submit a revision that we will return to the reviewers for re-evaluation.

We also noted the following points that need to be addressed.
- Abstract should include a clear concluding statement.
- Section numbering should be removed.
- Referencing is not in EJN style.
- Methods and results should not be split by experiment.
- Explicit statement of informed consent needed.
- Bar charts should be replaced with more informative scatter plots or similar (see Author Guidelines).

If you are able to respond fully to the points raised, we would be pleased to receive a revision of your paper within 30 days.

Thank you for submitting your work to EJN.

Kind regards,

Paul Bolam & John Foxe
co-Editors in Chief, EJN

Reviews:

Reviewer: 1 (Geoffrey Bird, University of Oxford, UK)

Comments to the Author
Review of EJN-2017-06-24646

I was incredibly impressed by this series of studies. In Study 1 the authors sought to replicate an initial effect of electrical stimulation of S1 on vicarious touch perception but to significantly improve the original method (number of trials, better control condition, bilateral stimulation) and extend the factors investigated (perspective, change of stimulation method). The authors found no effect of stimulation at all - despite a very good design - and as has been frequently noted we need to publish these null effects.

Experiment 2 followed the same aim but used tDCS (to more faithfully replicate the original report of stimulation effects) but extended the work to assess stimulation of TPJ and look at the effect of perspective. Again, no effects of stimulation were observed. The authors very carefully discuss these results and note

that the observed data is not consistent with their own theory - a practice rare but to be commended.

As noted, I was extremely impressed by these studies and the paper was written incredibly well. All analyses and designs were sound, and conclusions never strayed beyond the data. I highly recommend publication. However, I would like further comment on the use of on-line stimulation in Study 2. How did the use of online stimulation in Expt 2 compare to previous studies in this area (Coll et al, de Guzman et al, etc)? Could it explain the lack of replication?

Reviewer: 2 (Alessio Avananti, University of Bologna, Italy)

Comments to the Author
This is an interesting set of experiments examining the role of cortical excitability in somatosensory brain regions on vicarious tactile perception. In Experiment 1 the authors use active vs sham transcranial random noise stimulation to increase excitability in bilateral somatosensory regions, but find no difference between the conditions on vicarious tactile perception. In Experiment 2, the authors use tDCS in attempt to a) replicate prior findings that have shown that anodal tDCS can modulate vicarious tactile perception and b) extend them by considering additional factors related to task and brain region specificity that were not considered in prior. Again, the authors do not find strong evidence to suggest that vicarious tactile perception is modulated following tDCS to somatosensory regions. Although largely null results these findings have a number of theoretical implications for the field, which are well discussed. The direct replication of prior work in Experiment 2 is important and the authors provide a balanced discussion of potential factors contributing to differences in data between their own and others' findings. Overall I would say that the paper is of a high standard suitable for publication. There are some further features that the authors may wish to consider:

1. While the analyses are appropriate perhaps a Bayesian approach may also be useful. Particularly in Experiment 2 with regards to potential interactions between factors.
2. A number of studies point to the importance of baseline ability in modulating stimulation outcome. While the authors consider trait empathy, does baseline task performance (e.g. in the sham condition) relate to performance change following stimulation in a differential manner across conditions.
3. In prior work by one of the authors stimulation to right TPJ was conducted at 1mA and offline. This contrasts to experiment 2 where 1.5mA online stimulation is used. Presumably this was to match the somatosensory parameters that the author are replicating, but it might explain why the rTPJ stimulation did not modulate performance. Some discussion of this might be helpful.
4. I appreciate that the figures are probably the most useful way to depict the current results given the variety of factors investigated, but plotting individual performance data in addition to bar graphs might also be useful. Perhaps the authors could provide this in supplement materials.
5. The mechanism underlying somatosensory involvement in vicarious tactile perception is similar to those proposed for emotion and action perception. Interestingly, a few recent studies have shown that stimulation of the somatosensory cortex can affect perception of emotion expressions (e.g. Pitcher D et al., 2008, J Neurosci; Banissy M et al., 2010, J Neurosci; Paracampo R et al., 2016, Cereb Cortex) and actions (Jacquet P and Avenanti A, 2015, Cereb Cortex; Valchev N et al., 2017, Neuroimage). Yet, these sparse studies await replications and the present study – together with the evidence of large interindividual differences in the effects of brain stimulation – calls for more systematic investigation and replications in the field. This is important as there is an increasing interest in understanding the role of the somatosensory cortex in social perception. Authors may want to briefly consider these issues in revising their work.

Authors' Response 27 July 2017

RE: EJN-2017-06-24646

Thank you for taking the time to provide feedback on the previous version of our manuscript, and for recruiting two experts who have given detailed and useful comments. We were very grateful for the feedback we received. We were also pleased that you noted the rigour and high standard of the work, and that you would like to receive a revision of the manuscript for further consideration. In the revised version we believe that we have appropriately addressed all comments raised, leading to important improvements of the manuscript. In the reviewer response we detail how we have addressed each of the reviewer's comments (our response is shown in bold text). Our key additions include Bayesian statistical analyses of interactions most relevant to our replication attempt in Experiments 1 and 2, as was suggested by Reviewer 2. We also include additional graphical representations of individual variability in the effects of stimulation, requested by Reviewer 2 and the Editors, which we believe will be more informative than our original figures alone. We hope that you will now find our manuscript appropriate for publication, and look forward to hearing your response.

EJN European Journal of Neuroscience FENS Federation of European Neuroscience Societies

Yours Sincerely,

Natalie Bowling
(On behalf of both authors)

Comments from the editors and reviewers:

The Editors
Your manuscript was reviewed by two external reviewers and the process was handled by one of our Section Editors, Prof. Gregor Thut. Both reviewers commend your work for its rigour and high standard, and raise moderate to minor points for consideration, including additional statistics, analysis, Figures and discussion points. We believe you will be able to address these issues, and invite you to submit a revision that we will return to the reviewers for re-evaluation.
Thank you again for your feedback and positive comments on our manuscript. We found the feedback from reviewers and editors extremely useful for the improvement of the manuscript, and are pleased that we have been able to address all the issues raised.

We also noted the following points that need to be addressed.
- Abstract should include a clear concluding statement.
The abstract now includes a clear statement of the main conclusion of the two experiments: "Implications regarding mechanisms of vicarious perception are discussed. Collectively, the findings do not provide convincing evidence for the potential to modulate vicarious tactile perception with transcranial electrical current stimulation."

- Section numbering should be removed.
All section numbering has now been removed.

- Referencing is not in EJN style.
Referencing has been corrected to EJN style, including the abbreviation of journal titles in the reference list, and use of 'et al.' from the first instance for references with three or more authors.

- Methods and results should not be split by experiment.
The structure of the manuscript has now been adapted to a unified methods section for both experiments, and a results section which discusses the outcomes of Experiment 1 and 2 in turn. Discussion points from the interim summary and discussion, which was present in the original version of the manuscript, have now been incorporated into the general introduction and discussion sections, and are marked in bold on the revised manuscript.

- Explicit statement of informed consent needed.
The 'Participants' section of the methods (page 9) now includes an explicit statement of informed consent.

- Bar charts should be replaced with more informative scatter plots or similar (see Author Guidelines).
In our amended figures for both Experiments 1 and 2 (Figures 2 and 3) we now include plots of individual stimulation effects in each experimental condition. We believe that this will be more informative to readers interested in experimental effects at the individual level.

Reviewer 1
I was incredibly impressed by this series of studies. In Study 1 the authors sought to replicate an initial effect of electrical stimulation of S1 on vicarious touch perception but to significantly improve the original method (number of trials, better control condition, bilateral stimulation) and extend the factors investigated (perspective, change of stimulation method). The authors found no effect of stimulation at all - despite a very good design - and as has been frequently noted we need to publish these null effects.
Experiment 2 followed the same aim but used tDCS (to more faithfully replicate the original report of stimulation effects) but extended the work to assess stimulation of TPJ and look at the effect of perspective. Again, no effects of stimulation were observed. The authors very carefully discuss these results and note that the observed data is not consistent with their own theory - a practice rare but to be commended.

We thank Reviewer 1 for acknowledging the rigorous nature of our replication attempt, and for highlighting the importance of publishing null effects such as those we report in our manuscript.

As noted, I was extremely impressed by these studies and the paper was written incredibly well. All analyses and designs were sound, and conclusions never strayed beyond the data. I highly recommend publication. However, I would like further comment on the use of on-line stimulation in Study 2. How did the use of online stimulation in Expt 2 compare to previous studies in this area (Coll et al, de Guzman et al, etc)? Could it explain the lack of replication?
Thank you for raising this important consideration, which we now discuss in our manuscript. Our decision to use 1.5mA online stimulation was in order to ensure that the same stimulation parameters were used for somatosensory and rTPJ stimulation. We selected 1.5mA online stimulation because this was what was used to stimulate the somatosensory cortex in Bolognini et al. (2013): our primary aim was to attempt to replicate the Bolognini study. You are, however, correct that the tDCS parameters used to stimulate rTPJ in our experiment are different to work of Coll et al (2017) and some other work in the literature (e.g. Santiesteban et al. 2012, 2015). This could provide an additional explanation of why effects of stimulation targeted at rTPJ were not significant. We have added a brief comparison of our methods with those of previous studies to the discussion section of the manuscript, and raise this issue as a potential explanation of our null results:
"It should be noted that the tDCS parameters in the present study differed from those used in previous experiments modulating activity of rTPJ with tDCS. For instance, prior work examining self-other representation using tDCS targeted at rTPJ has stimulated offline at 1mA using 5x7 cm electrodes (e.g. Santiesteban et al., 2012; Santiesteban et al., 2015a; Sowden et al., 2015) or 5x7 cm and 10x10 cm electrodes (e.g. Liepelt et al., 2016). Similarly, Coll and colleagues (2017) used 2mA tDCS with 5x7cm electrodes to assess vicarious pain perception. Our decision to use a protocol involving online stimulation at 1.5mA using 5x5cm electrodes was selected to match that in the rSI stimulation condition (which was selected to replicate prior work - Bolognini et al., 2013). The differences between our rTPJ stimulation montage and those used in other studies may have affected the degree of modulation of rTPJ compared with past research. This could account for the lack of influence of rTPJ stimulation on vicarious perception in Experiment 2."

Reviewer 2
This is an interesting set of experiments examining the role of cortical excitability in somatosensory brain regions on vicarious tactile perception. In Experiment 1 the authors use active vs sham transcranial random noise stimulation to increase excitability in bilateral somatosensory regions, but find no difference between the conditions on vicarious tactile perception. In Experiment 2, the authors use tDCS in attempt to a) replicate prior findings that have shown that anodal tDCS can modulate vicarious tactile perception and b) extend them by considering additional factors related to task and brain region specificity that were not considered in prior. Again, the authors do not find strong evidence to suggest that vicarious tactile perception is modulated following tDCS to somatosensory regions. Although largely null results these findings have a number of theoretical implications for the field, which are well discussed. The direct replication of prior work in Experiment 2 is important and the authors provide a balanced discussion of potential factors contributing to differences in data between their own and others' findings. Overall I would say that the paper is of a high standard suitable for publication. There are some further features that the authors may wish to consider:
We would like to thank Reviewer 2 for the positive comments on our manuscript, including our discussion of the theoretical implications of the work. We are delighted that Reviewer 2 also recommends the paper for publication.

1. While the analyses are appropriate perhaps a Bayesian approach may also be useful. Particularly in Experiment 2 with regards to potential interactions between factors.
We agree that a Bayesian approach to the statistical analysis of our data would be useful here for quantifying the evidence in favour of the null hypothesis, particularly for the interactions which fail to replicate in our experiments. Bayesian analyses for both Experiments 1 and 2 have been added to the revised manuscript.

2. A number of studies point to the importance of baseline ability in modulating stimulation outcome. While the authors consider trait empathy, does baseline task performance (e.g. in the sham condition) relate to performance change following stimulation in a differential manner across conditions.

We share the view that baseline ability is an important consideration in studies which attempt to modulate performance with non-invasive brain stimulation. Prior to writing the original manuscript we chose to examine these effects with a correlation and regression analyses to identify whether baseline performance related to performance change following stimulation, with the prediction that participants with lower levels of vicarious perception would show a greater increase with stimulation of SI. For this analysis we used congruency effects (the difference between reaction times on congruent and incongruent trials) as a measure of vicarious perception, and these were entered as a predictor of stimulation effects (the difference between congruency effects under sham and stimulation conditions). The analysis showed a significant negative relation between the variables, under all tasks and stimulation conditions. That is to say that there was a non-specific effect of baseline (i.e. the effect did not distinguish between conditions). Due to the lack of specificity of the effects, we interpreted this as reflecting regression to the mean. According to this phenomenon it is expected that extreme scores will tend towards the mean on a second measurement – i.e. individuals with lower than average congruency effects at sham will increase, and individuals with higher than average effects will decrease, as was observed. For this reason, we decided not to include this analysis in the manuscript, as it is difficult to isolate true individual difference effects from regression to the mean. We agree that this will be an interesting line of investigation and are planning to pursue it in future work.

3. In prior work by one of the authors stimulation to right TPJ was conducted at 1mA and offline. This contrasts to experiment 2 where 1.5mA online stimulation is used. Presumably this was to match the somatosensory parameters that the author are replicating, but it might explain why the rTPJ stimulation did not modulate performance. Some discussion of this might be helpful.
This point is an important one and was also raised by Reviewer 1. In view of your comments and those raised by reviewer 1 we now state the following in our discussion:

"It should be noted that the tDCS parameters in the present study differed from those used in previous experiments modulating activity of rTPJ with tDCS. For instance, prior work examining self-other representation using tDCS targeted at rTPJ has stimulated offline at 1mA using 5x7 cm electrodes (e.g. Santiesteban et al., 2012; Santiesteban et al., 2015a; Sowden et al., 2015) or 5x7 cm and 10x10 cm electrodes (e.g. Liepelt et al., 2016). Similarly, Coll and colleagues (2017) used 2mA tDCS with 5x7cm electrodes to assess vicarious pain perception. Our decision to use a protocol involving online stimulation at 1.5mA using 5x5cm electrodes was selected to match that in the rSI stimulation condition (which was selected to replicate prior work - Bolognini et al., 2013). The differences between our rTPJ stimulation montage and those used in other studies may have affected the degree of modulation of rTPJ compared with past research. This could account for the lack of influence of rTPJ stimulation on vicarious perception in Experiment 2."

4. I appreciate that the figures are probably the most useful way to depict the current results given the variety of factors investigated, but plotting individual performance data in addition to bar graphs might also be useful. Perhaps the authors could provide this in supplement materials.
Thank you for acknowledging that our original figures were useful. The original figures that we included were selected to provide the simplest depiction of our complex data set, with several conditions to compare. However, we agree that some representation of individual performance data would be beneficial. Our revised figures include plots of individual stimulation effects in each experimental condition, which we hope will be informative to the reader.

5. The mechanism underlying somatosensory involvement in vicarious tactile perception is similar to those proposed for emotion and action perception. Interestingly, a few recent studies have shown that stimulation of the somatosensory cortex can affect perception of emotion expressions (e.g. Pitcher D et al., 2008, J Neurosci; Banissy M et al., 2010, J Neurosci; Paracampo R et al., 2016, Cereb Cortex) and actions (Jacquet P and Avenanti A, 2015, Cereb Cortex; Valchev N et al., 2017, Neuroimage). Yet, these sparse studies await replications and the present study – together with the evidence of large interindividual differences in the effects of brain stimulation – calls for more systematic investigation and replications in the field. This is important as there is an increasing interest in understanding the role of the somatosensory cortex in social perception. Authors may want to briefly consider these issues in revising their work.
This is a very interesting point of discussion, which highlights the wider implications of our present results to the mechanisms underlying social perception and the need for further replication studies in this area. We have added the following comments to the discussion section:

EJN European Journal of Neuroscience FENS Federation of European Neuroscience Societies

“It is also important to consider how the present findings relate to the broader literature regarding the role of sensorimotor contributions to social perception. For instance, recent evidence has indicated that a range of social perception abilities are linked with sensorimotor cortex activity (e.g. Adolphs et al., 2000; Banissy et al., 2010; Jacquet & Avenanti, 2015; Keysers et al., 2010; Paracampo et al., 2016; Pitcher et al., 2008; Pourtois et al., 2004; Valchev et al., 2017). Several of these have used non-invasive brain stimulation to show changes in social perception skills following sensorimotor cortex stmulation relative to appropriate control conditions (e.g. baseline, control brain stimulation conditions). There have, however, been few published replication attempts for these studies. The present study, together with the evidence of large inter-individual differences in the effects of non-invasive brain stimulation (e.g. Fertonani & Miniussi, 2017; Hsu et al., 2016; Ridding & Ziemann, 2010), calls for more systematic investigations and replications in this area.”
